# Biogenic Imaging Contrast Agents

**DOI:** 10.1002/advs.202207090

**Published:** 2023-07-03

**Authors:** Qing Dan, Xinpeng Jiang, Run Wang, Zhifei Dai, Desheng Sun

**Affiliations:** ^1^ Shenzhen Key Laboratory for Drug Addiction and Medication Safety Department of Ultrasound Institute of Ultrasonic Medicine Peking University Shenzhen Hospital Shenzhen Peking University‐The Hong Kong University of Science and Technology Medical Center Shenzhen 518036 P. R. China; ^2^ Department of Biomedical Engineering College of Future Technology Peking University Beijing 100871 P. R. China

**Keywords:** biogenic, contrast agent, imaging, reporter gene

## Abstract

Imaging contrast agents are widely investigated in preclinical and clinical studies, among which biogenic imaging contrast agents (BICAs) are developing rapidly and playing an increasingly important role in biomedical research ranging from subcellular level to individual level. The unique properties of BICAs, including expression by cells as reporters and specific genetic modification, facilitate various in vitro and in vivo studies, such as quantification of gene expression, observation of protein interactions, visualization of cellular proliferation, monitoring of metabolism, and detection of dysfunctions. Furthermore, in human body, BICAs are remarkably helpful for disease diagnosis when the dysregulation of these agents occurs and can be detected through imaging techniques. There are various BICAs matched with a set of imaging techniques, including fluorescent proteins for fluorescence imaging, gas vesicles for ultrasound imaging, and ferritin for magnetic resonance imaging. In addition, bimodal and multimodal imaging can be realized through combining the functions of different BICAs, which helps overcome the limitations of monomodal imaging. In this review, the focus is on the properties, mechanisms, applications, and future directions of BICAs.

## Graphical abstract

Biogenic imaging contrast agents (BICAs) facilitate various studies, such as quantification of gene expression, observation of protein interactions, visualization of cellular proliferation, monitoring of metabolism, and detection of dysfunctions. Matched with a set of imaging techniques, including fluorescent proteins for fluorescence imaging, gas vesicles for ultrasound imaging, and ferritin for magnetic resonance imaging, BICAs can realize diseases detection and monitoring.

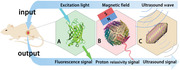

## 1 Introduction

Visualization of location and function of specific cells and molecules inside the body is critical for diseases detection and monitoring as well as basic biological studies of cellular and genetic therapeutics. There are various well‐established imaging modalities, classified as non‐radiative imaging, such as optical fluorescence (FL) imaging, ultrasound (US) imaging, and magnetic resonance imaging (MRI); and radiative imaging, such as computed tomography (CT), positron emission computed tomography (PET), and single‐photon emission computed tomography (SPECT). Compared to conventional imaging, the contrast agents enhanced imaging techniques push forward the evolution of anatomy imaging into functional and molecular imaging. Non‐radiative imaging techniques are likely to combine with a variety of endogenous biogenic imaging contrast agents (BICAs) to achieve cell‐based diagnostic imaging, such as gene expression, proteins interaction, and cell migration. In contrast, radiation‐based imaging modalities require exogenously administered contrast agents.^[^ ^1^, ^2^, ^3^ ^]^ Hence, this review focuses on non‐radiative imaging, including FL imaging, US imaging, and MRI.

**Figure 1:**
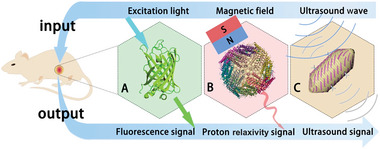
Schematic illustration of BICAs. A) Fluorescent protein for FL imaging. FL imaging requires materials that can emit fluorescent signals under excitation of light source. B) GV for US imaging employs materials that can reflect sound waves for imaging based on acoustic impedance between tissues. C) Ferritin for MRI. MRI utilizes materials which are able to interact with surrounding nuclear spins.

Conventionally synthesized contrast agents are useful for the enhanced contrast imaging at macro level based on body fluid flow. However, they are incapable of visualizing specific cellular functions and metabolism at micro level.^[^ ^4^, ^5^, ^6^ ^]^ Instead, endogenously generated or genetically engineered BICAs are designed to image the functions of specific proteins and intracellular interactions in vivo for better diseases detection and monitoring.^[^ ^7^, ^8^ ^]^ We will discuss three types of BICAs (**Figure** **1**), such as fluorescent proteins for FL imaging, gas vesicles (GVs) for US imaging, and ferritin for MRI, elaborating the properties, mechanisms, applications, and future directions of BICAs, with a focus on their abilities of imaging the function of specific cells and molecules inside living organisms. In addition, the potential applications of bimodal imaging with BICAs will be covered.

## 2 Principles of Main Non‐Invasive Imaging Modalities

A non‐invasive and non‐radiative assessment of the invisible in vivo biological processes advances the development of basic biological research and cellular and genetic therapeutics. FL imaging, US imaging, and MRI covered in this review are playing great roles in visualizing physiologic functions and pathological conditions of living systems.

**Figure 2:**
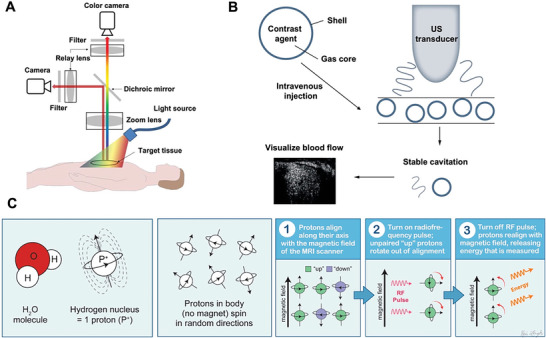
A) Schematic diagram of an FL imaging system. Chromophores in target tissues emit FL signals when excited by light source. Reproduced under the terms of the Creative Commons CC‐BY license.^[^ ^19^ ^]^ Copyright 2022, The Authors. Published by BMJ. B) Schematic illustration of administrated US contrast agents enhancing the echogenic signals of the blood pool. Contrast agents, such as microbubbles, in blood vessels reflect the signals of sound waves; thus; increasing the brightness (echogenicity) compared to tissues where there are no microbubbles. Reproduced under the terms of the Creative Commons CC‐BY license.^[^ ^20^ ^]^ Copyright 2020, The Authors. Published by Future Science. C) Schematic illustration of mechanism of MRI. RF, radiofrequency. Reproduced under the terms of the Creative Commons CC‐BY license.^[^ ^21^ ^]^ Copyright 2021, The Authors. Published by MDPI.

In brief, FL imaging can visualize subcellular process with high resolution and sensitivity but exhibits strong light scattering and limited light penetration (**Figure** **2**A). Various biomolecular agents, including fluorescent proteins (FPs),^[^ ^9^ ^]^ fluorogenic RNAs,^[^ ^10^ ^]^ protoporphyrin IX (PpIX),^[^ ^11^ ^]^ and melanin,^[^ ^12^ ^]^ are mostly used for FL imaging.^[^ ^13^ ^]^ Exogenously, FPs and fluorogenic RNAs could be integrated into a colorful toolbox for biomedical research, while PpIX and melanin are usually utilized for cancer research due to their unique characteristics toward tumor metabolism. Contrast‐enhanced US imaging utilizes microbubbles to amplify the ultrasonic reflection in blood vessels with excellent depth penetration (Figure 2B),^[^ ^14^ ^]^ which is generally applied to myocardial perfusion imaging and differentiation of benign and malignant lesions, however, without demonstrating the subcellular information. GVs as gas‐filled protein nanostructures that are detectable at subnanomolar concentrations can serve as highly sensitive imaging agents for US and MRI to visualize the dynamic behaviors and functions of cells. GVs can not only be chemically modified to target cell surface receptors but also be genetically encoded to express as a reporter gene in cells.^[^ ^15^ ^]^ MRI uses magnets and radio waves to generate images of almost every structure and organ inside the body. It is a powerful tool to non‐invasively observe the function of specific cells and molecules within the context of living organisms with high spatiotemporal resolution (Figure 2C). However, the intrinsic toxicities of chemically synthetic contrast agents, such as gadolinium‐based agents, limit their biomedical applications. Biogenic alternatives inherently generated from the body can enhance contrast in MRI. The most classical imaging methods are relaxation time‐weighted imaging (TWI) that uses metalloprotein and ferritin as the contrast agents and chemical exchange saturation transfer (CEST) MRI that observes endogenous proteins and metabolites with exchangeable protons.^[^ ^16^ ^]^ Currently, biomolecular reporters such as aquaporin (AQP) for diffusion‐weighted imaging (DWI) and GVs for hyperpolarized CEST (HyperCEST) MRI are increasingly reported.^[^ ^17^, ^18^ ^]^

## 3 BICAs and Their Applications

In medical imaging, contrast agents are a set of substances used to increase the contrast of structures or fluids within the body. These agents have their own unique properties and different interactions with light, US wave, and magnetic field; so that, they are used to enhance the contrast of these imaging modalities. Although each BICA for each imaging modality does not share common physical properties, chemical structures, and imaging mechanisms, they show some similar advantages for observation of in vivo proteins and cells in an efficient and safe manner. For example, BICAs could detect endogenous substances at low concentrations, which is unlikely to occur in conventional imaging techniques. In addition, BICAs can be readily engineered due to their intrinsic properties compared to existing contrast agents. Their characteristics and performance will be discussed thoroughly below.

### 3.1 FL Imaging Contrast Agents

#### 3.1.1 Fluorescent Proteins (FPs)

Some jellyfish species emit light from different parts of their bodies in the dark ocean, which helps them to warn off predators. This process of glowing results in the discovery of epoch‐making FP from the Pacific Northwest jellyfish *Aequorea victoria*, which is known as *Aqueous victoria* green FP (avGFP), launching a new era of a FL marker for gene expression and localization of gene products.^[^ ^22^ ^]^ Genetically encoding GFP molecule of invisible proteins allows visualization of the movement, degradation, and synthesis of these proteins. Hence, many protein malfunctions associated with several illnesses and diseases can be detected.^[^ ^23^ ^]^

**Figure 3:**
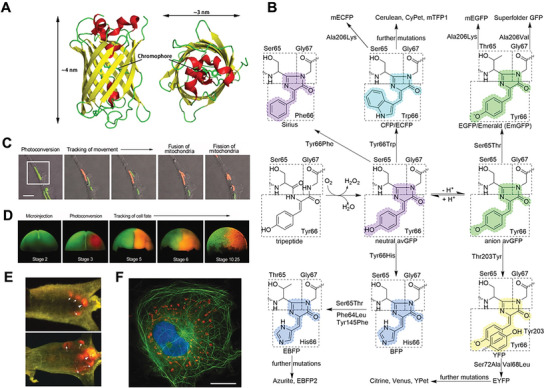
A) avGFP *β*‐barrel architecture and approximate dimensions (drawing was based on Protein Data Bank ID: 1W7S). Left: front view; right: top view. 11 stranded *β*‐barrel fold structure is colored with yellow. B) Mutant relationships, chromophores, and important mutation sites of GFP‐like proteins. C) Mitochondria were labeled using td‐EosFP. White rectangle: a single mitochondrion photoconverted from green to red by irradiation with 405 nm light. Scalebar: 1 µm. D) Cell tracking multicolor during early embryonic development of Xenopus laevis. E) In vivo whole‐body imaging of tumor progression in nude mice using DsRed‐2. White arrowhead: primary tumor; white arrows: metastases. F) imaging in HeLa cells. Green: EGFP‐labeled tubulin‐associated protein; red: mitochondrial RFP611; blue: DAPI. Scalebar: 10 µm. (C–F) Reproduced under the terms of the Creative Commons CC‐BY license.^[^ ^23^ ^]^ Copyright 2009, The Authors. Published by IUBMB.

Biological imaging applications of FPs result from their unique chemical structures. FPs originate from a structurally homologous class of proteins that self‐sufficiently forms a visible wavelength chromophore from a sequence of three amino acids within their own polypeptide sequence. GFPs consist of 238 amino acids with the molecular weight of about 25 kDa and exhibit the classic 11‐stranded *β*‐barrel fold structure (**Figure** **3**A). This kind of barrel fold structure is adopted by other FPs to anchor and protect the chromophore in the core, which is responsible for the strong FL emission.^[^ ^24^ ^]^ The chromophore of GFP itself is a 4‐(*p*‐hydroxybenzylidene)−5‐imidazolinone (*p*‐HBI), consisting of residues 65–67 (Ser‐*dehydro*Tyr‐Gly) of the protein and exhibiting green FL. Considerable GFP‐like proteins with improved properties and different spectrums are generated from genetic engineering of GFPs by site‐directed and random mutation on and around the chromophore's amino acids (Figure 3B). High‐performance variants of avGFP with emission peaks ranging from blue to yellow,^[^ ^25^ ^]^ red‐shift FPs,^[^ ^26^ ^]^ and near‐infrared FPs (NIR FPs),^[^ ^27^ ^]^ were developed and refined dramatically for biological studies over the two decades.

As an FL imaging contrast agent, FP facilitates labeling of organelle inside cells (Figure 3C), marking embryonic cells to visualize embryo development (Figure 3D),^[^ ^28^, ^29^ ^]^ monitoring tumor metastasis (Figure 3E), and enabling FL‐guided surgery.^[^ ^30^, ^31^ ^]^ In addition, FPs with wide spectrum of color enable the observation of multiple subcellular structures simultaneously through multicolor labeling (Figure 3F). Currently, the main issue of in vivo FL imaging lies in the limited tissue penetration; hence, vast demand exists to improve the imaging performance within the context of intact organisms by developing longer wavelength emissive NIR FPs.

#### 3.1.2 NIR FPs

**Figure 4:**
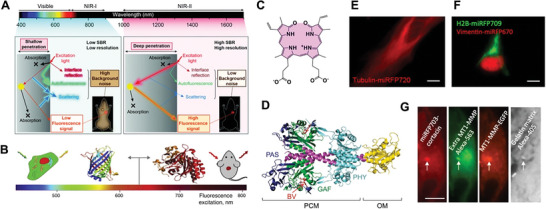
A) Schematic illustration of the FL signals with different wavelengths in biological tissues. SBR, signal‐background ratio. Reproduced under the terms of the Creative Commons CC‐BY license.^[^ ^32^ ^]^ Copyright 2020, The Authors. Published by Royal Society of Chemistry. B) FPs of GFP family and NIR FPs with different emission wavelengths. Adapted with permission.^[^ ^44^ ^]^ Copyright 2015, ScienceDirect. C) Chemical structure of BV. D) Structure of BphP including PAS, GAF, and PHY domain. Adapted with permission.^[^ ^27^ ^]^ Copyright 2019, Springer Nature. E) Tubulin‐miRFP720 localizes well in live HeLa cells. Scalebar: 10 µm. F) Two‐color labeling using miRFP670 and miRFP709 allows simultaneous visualization of H2B and vimentin in HeLa cells. Scalebar: 10 µm. G) Four‐color imaging to localize invadopod (miRFP703), extracellular (Alexa‐565) MT1‐matrix metalloproteinase, intracellular (EGFP), and matrix degradation (Alexa‐405). Scalebar: 20 µm. (E–G) Adapted with permission.^[^ ^43^ ^]^ Copyright 2018, Elsevier.

Compared to imaging with traditional visible FPs, imaging in the NIR window of the spectrum (≈650–900 nm) with NIR FPs overcomes the limitations of shallow tissue penetration of visible light.^[^ ^32^ ^]^ Therefore, the FL imaging of living organisms shows higher signal‐background ratio due to less tissue absorbance and scattering, as well as low autofluorescence and reduced phototoxicity for living cells (**Figure** **4**A,B). Thus, NIR FPs are extremely useful for imaging ranging from individual molecules to whole organisms.

NIR FPs engineered from bacterial phytochromes (BphPs) have been commonly used for non‐invasive in vivo imaging to observe functional activities ranging from molecular to organismal levels. BphPs use linear tetrapyrrole compounds bilins as the chromophores.^[^ ^33^ ^]^ One of the farthest red‐shift bilins is biliverdin IX*α* (BV)^[^ ^34^ ^]^ (Figure 4C), which widely exists in BphPs^[^ ^35^ ^]^ (Figure 4D). Being a metabolite of heme oxygenase, BV is also ubiquitous in many eukaryotic organisms including flies, fishes, and mammals,^[^ ^36^ ^]^ making BphP‐based FPs applicable in live mammals for whole‐body FL imaging.^[^ ^37^, ^38^ ^]^ However, endogenous BV varies in different organs and enriches in liver and spleen; therefore, the imaging of NIR FPs has not been demonstrated in BV‐lacked tissues.

The development of NIR FPs was mainly based on the evolution of the *Deinococcus radiodurans* BphP (DrBphP)^[^ ^27^ ^]^ and *Rhodopseudomonas palustris* BphP (RpBphP).^[^ ^9^ ^]^ iRFP713, one of the first reported NIR FPs, is now regarded as a reference standard for newly developed NIR FPs.^[^ ^9^ ^]^ Up to date, various NIR FPs, including dimeric iRFP670, iRFP682, iRFP702, iRFP720, and monomeric miRFP670, miRFP703, and miRFP709,^[^ ^39^, ^40^, ^41^ ^]^ can be used in monomeric and multicolor imaging. For example, miRFPs as versatile fusion tags localize well in protein fusions, which can be utilized as reporters and biosensors of biological processes within the context of living organisms (Figure 4E). Moreover, two‐color labeling employing miRFP670 and miRFP709 enables simultaneous visualization of H2B and vimentin in HeLa cells (Figure 4F). In addition, four‐color imaging using miRFP703 illustrates the mechanism of breast cancer invasion and metastasis associated with activity of Rac3 GTPase localized at invadopodia, which mediates the degradation of extracellular matrix, promoting cancer cell invasion (Figure 4G).^[^ ^42^ ^]^

Generally, compared to GFP‐like FPs, NIR FPs have a much lower fluorescent quantum yield, hindering the full potential of NIR FL imaging with deep penetration and high temporal resolution. Further efforts are required to address this issue by protein engineering technology, novel precursor bacterial phytochromes for evolution, and advanced imaging modality.^[^ ^43^ ^]^ In addition, the development of NIR FPs with further red‐shift spectra facilitates deeper tissue penetration and lower autofluorescence background for in vivo imaging.

#### 3.1.3 Fluorogenic RNAs

The development of FPs has led to the emergence of molecular imaging in the realm of RNA. The RNA mimics of FPs are molecules, termed as aptamers, that recognize and subsequently bind to cognate small molecules. Fluorogenic RNA aptamers enhance the FL by as much as 5000 times of an unbound fluorophore that is otherwise minimally fluorescent on its own,^[^ ^10^ ^]^ allowing the visualization of RNA molecules within a cell.

**Figure 5:**
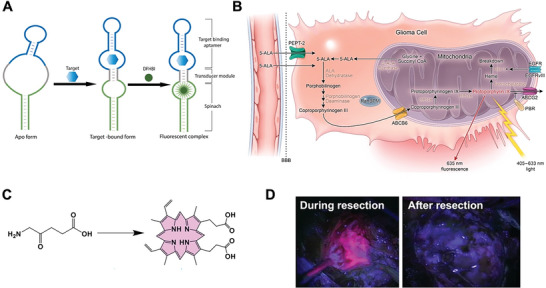
A) Schematic of allosteric Spinach. In the presence of target (e.g., metabolites), target‐binding aptamer folds and induces the correct folding of Spinach through a transducer module. Last, DFHBI binds with Spinach to produce a fluorescent complex. Adapted with permission.^[^ ^60^ ^]^ Copyright 2019, ScienceDirect. B) Heme synthesis pathway and mechanism of 5‐ALA FL. ABCG2, ATP‐binding cassette G2; ABCB6, ATP‐binding cassette B6; BBB: blood‐brain barrier; CPOX, coproporphyrinogen oxidase; HO‐1: heme‐oxygenase 1; PBR: peripheral benzodiazepine receptor; PPOX: protoporphyrinogen oxidase. Reproduced under the terms of the Creative Commons CC‐BY license.^[^ ^55^ ^]^ Copyright 2021, The Authors. Published by MDPI. C) The conversion from 5‐ALA to PpIX in tumor cells. D) FL‐guided surgery of a high‐grade glioma using 5‐ALA. Adapted with permission.^[^ ^58^ ^]^ Copyright 1998, Wolters Kluwer Health.

The first reported FL turn‐on RNA aptamer is termed as Spinach. Its chromophore (3,5‐difluoro‐4‐hydroxybenzylidene imidazolinone (DFHBI)) is derived from HBI, the chromophore of GFP;^[^ ^45^ ^]^ hence, Spinach is considered as the RNA mimic of GFP. DFHBI exhibits very low FL in its free form, while it can exhibit strong green FL by about 2000 folds in complex with Spinach (**Figure** **5**A).^[^ ^46^ ^]^ Due to its FL turn‐on property and excellent brightness, Spinach demonstrates the potential of an aptamer–fluorophore complex, serving as a fluorescent tag for in vivo imaging of cellular RNAs. For example, Spinach was reported being able to monitor transcription in vitro as well as successfully being utilized as a cell RNA‐imaging tag by expressing ribosomal RNA–Spinach fusions in mammalian cells.^[^ ^10^ ^]^

Nevertheless, Spinach shows several limitations, such as misfolding inside cells, low thermostability, and sensitivity to flanking sequences.^[^ ^46^ ^]^ Several Spinach RNA mutations, including Spinach2,^[^ ^47^ ^]^ Baby Spinach,^[^ ^48^ ^]^ and iSpinach,^[^ ^49^ ^]^ have been developed to improve the shortcomings of Spinach. A more popular and favorable fluorogenic RNA is Broccoli, which consists of 49 nucleotides and has a simplified cellular folding condition.^[^ ^50^ ^]^ In addition to Spinach and Broccoli, other groups of fluorogenic RNAs, including Mango,^[^ ^51^ ^]^ DNB,^[^ ^52^ ^]^ Corn,^[^ ^53^ ^]^ and SRB‐2,^[^ ^54^ ^]^ can activate various kinds of fluorophores, which allow target detection at different wavelengths.

#### 3.1.4 5‐Aminolevulinic Acid (5‐ALA)

The 5‐ALA is a natural amino acid and a porphyrin precursor in the heme‐biosynthesis‐pathway. Certain cancers, such as gliomas, liver cancer, and bladder cancer, take up exogeneous 5‐ALA and convert it to the fluorogenic metabolite PpIX in the mitochondria. After combining with iron, regulated by the enzyme ferrochelatase, PpIX accumulation can be visualized as red FL under illumination by blue–violet light (Figure 5B,C).^[^ ^55^ ^]^ Of note, in normal cells, exogeneously supplied 5‐ALA is rapidly metabolized to heme. In contrast, in cancer cells, due to alteration of enzymes activity and/or 5‐ALA influx transporter in the heme synthesis pathway, the heme precursor PpIX is preferentially accumulated.^[^ ^56^ ^]^ However, the molecular and metabolic mechanisms of selective accumulation of PpIX in cancer cells need further elucidation. Currently, the well acknowledged hypotheses are CPOX upregulation and ferrochelatase downregulation in the heme synthesis, and increased cell density as well as distortion of blood–brain‐barrier during the development of malignancies.^[^ ^57^ ^]^

In the initial study reported by Stummer and coworkers in 1998, 5‐ALA‐induced tissue FL was used for intraoperative tumor tissue discrimination of high grade glioma at high sensitivity, specificity, and accuracy.^[^ ^58^ ^]^ Normal brain tissue revealed no porphyrin FL, whereas tumor tissue was specifically distinguished by bright red FL. As shown in Figure 5D, the glioma tissue exhibited strong red FL during surgery while no obvious FL was observed after resection. Based on further findings,^[^ ^55^, ^59^ ^]^ 5‐ALA is well accepted for FL‐guided resection of high grade gliomas because it significantly improves the tumor removal rate and patient outcomes, and it is readily bioavailable after oral administration and rapidly excreted out from the body within 24 h.

Despite 5‐ALA being a powerful tool for FL‐guided surgery, there remain several challenges including elimination of background autofluorescence and non‐selective accumulation of PpIX in non‐tumor tissues, such as inflammatory regions. Currently, sophisticated optical approaches such as real‐time FL lifetime imaging (FLIM) have been used to enhance the contrast of weakly fluorescent tissues, decreasing the impact of tissue‐derived autofluorescence.^[^ ^56^ ^]^ In addition, further studies on the mechanism of PpIX accumulation are required to reduce false positive induced by PpIX accumulation in non‐tumor tissues.

#### 3.1.5 Melanin

Melanin is a natural amino acid derivative produced and stored by melanocytes through a series of chemical reactions from tyrosine and 3,4‐dihydroxyphenylalanine. If the metabolism of melanocytes is destroyed or inhibited, some diseases will occur, such as genetic diseases albinism and melanoma.^[^ ^61^ ^]^ Spectral analysis of melanoma revealed a main FL peak around 470 nm with an additional peak close to 550 nm throughout all epidermal layers,^[^ ^62^ ^]^ allowing melanin to serve as a good endogenous cancer marker. Based on its distinct characteristic, there are some studies on the FL diagnosis of melanoma. Procedures of selective imaging as well as spectral FLIM through multiphoton laser tomography (MPT) support diagnostic decisions and may improve the process of non‐invasive early detection of melanoma.^[^ ^62^ ^]^ By combining FLIM with MPT, Stefania Seidenari et al. achieved a non‐invasive investigation of the skin within subcellular resolution.^[^ ^63^ ^]^

More commonly, melanin is used in PAI because it has highly sensitive optical absorption and heat expansion; and then, stimulates acoustic waves of the surrounding medium for deep tissue imaging.^[^ ^13^ ^]^ It has absorption of 1000 folds than that of water at 700 nm wavelength; hence, the significant contrast between melanoma and background tissue can be achieved using PAI.^[^ ^64^ ^]^ Furthermore, the circulating melanoma cells with overexpressed melanin have been detected with high contrast to blood background.^[^ ^65^, ^66^, ^67^ ^]^ Due to the good light‐to‐heat conversion capability, melanin is also fabricated as a photothermal therapy (PTT) agent to achieve PAI guided PTT.^[^ ^68^, ^69^, ^70^ ^]^ More efficiently, the natural polymer melanin can be functionalized into drug‐loaded nanoparticles to realize co‐delivery of chemotherapy drug^[^ ^68^ ^]^ and gene therapeutic agents^[^ ^69^ ^]^ and achieve synergistic therapy with integrated monitoring.

## 4 GVs for US Imaging

The biomedical applications of current fluorescent molecular biosensors are limited by the tissue penetration depth while US imaging can easily overcome this restriction with deep tissue penetration ability. Conventional US contrast agents are micro‐sized gas bubbles, termed as microbubbles, which are encapsulated by a biocompatible shell, such as proteins, lipids, and polymers.^[^ ^71^ ^]^ In clinical diagnosis, microbubbles‐assisted US imaging is primarily used within vasculatures. In addition, considerable preclinical studies have demonstrated that microbubbles functionalized with specific ligands enable imaging of certain molecular targets, including tumors and vasculatures.^[^ ^72^ ^]^ However, microbubbles face restrictions as suitable molecular reporters owing to their large size and physical instability. US imaging has not reached its great potential in biomedical research at cellular and genetic level. However, biogenic US contrast agents, such as GVs produced by microorganism, have increasingly emerged to bridge the gap between US imaging and biomolecular and cellular activities inside living organisms.

### 4.1 GVs

GVs are unique gas‐filled and protein‐shelled nanostructures, which are expressed in a range of bacteria and archaea to regulate cellular buoyancy for growth in watery environments. GVs are mostly found in cyanobacteria, cold‐loving heterotrophic bacteria such as *Ancylobacter aquaticus*, anoxygenic photosynthetic bacteria such as *Anabaena flos‐aquae* (*Dolichospermum flos‐aquae*), and mesophilic haloarchaea such as *Halobacterium salinarum*.^[^ ^73^ ^]^

**Figure 6:**
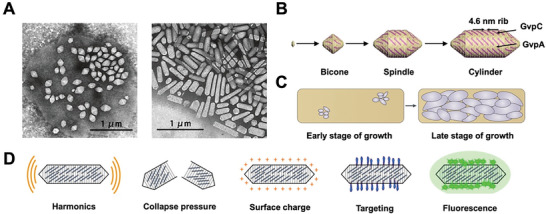
A) Transmission electron microscopy (TEM) of a lysed *H. salinarum* cell containing spindle‐shaped GVs (left). Isolated cylinder‐shaped GVs of *H. salinarum* (right). B) Morphogenesis of GVs start from aggregated proteins that form a bicone; then, grow to form spindle‐ or cylinder‐shape. GvpA forms 4.6 nm‐wide ribs and runs nearly perpendicular to the long axis of the GV. GvpC stabilizes the GV wall by attaching to the exterior surface. C) During the early stages of GVs formation, only a few small GVs are produced in cells; and later, on cells are filled with large GVs. (A–C) Adapted with permission.^[^ ^73^ ^]^ Copyright 2012, Springer Nature. D) Genetical modification of GvpC modulates the properties of GVs, including harmonic signals, collapse pressure, surface charge, targeting ability, and fluorescent signals. Adapted with permission.^[^ ^76^ ^]^ Copyright 2018, American Chemical Society.

A single cell might contain a few to more than a hundred GVs. GVs start from aggregated bicone‐like proteins and later on grow to become spindle‐ or cylinder‐shaped, depending on genetic hosts and environmental conditions (**Figure** **6**A–C). Generally, GVs have a width of around 100–250 nm and a length of up to 2 µm. Their shell, with a thickness of about 2 nm, is only formed by a small hydrophobic GV protein (GvpA, molecular weight of about 7–8 kDa) to form a single layer “rib” wall and stabilized by a larger hydrophilic protein (GvpC, molecular weight of appro 31–42 kDa).^[^ ^73^ ^]^ Unlike microbubbles, which load gas in an unstable manner, GVs exclude surrounding water and allow gas to freely permeate in and out to maintain an equilibrium via small holes on their protein shells, making them physically stable.

GV proteins are required as auxiliary structural proteins, chaperones, or as gene regulators. Regulatory proteins include negative regulatory protein—GvpD and transcriptional activator—GvpE, where the regulation occurs mostly at transcription stage, sometimes at translation level. Possible minor structural proteins or assembly proteins are GvpF and GvpG. Some proteins, GvpI and GvpN, are associated with the sizes and shapes.^[^ ^74^ ^]^ Deletion of *gvp*D and *gvp*E leads to reduction of GVs amounts, whereas deletion of *gvp*H or *gvp*C leads to fragile or large and egg‐shaped GVs.^[^ ^74^ ^]^ Genetic engineering of GvpC to generate GVs with multiple collapse pressures was also used to produce a series of GVs with broad acoustic characteristics which further broadens the range of US responses.^[^ ^75^ ^]^

As mentioned above, the shape of GVs is determined by genetic hosts and environmental conditions. Totally, about 8–14 different Gvps encoded in *gvp* gene clusters are involved in GVs formation (**Table** **1**). In *H. salinarum*, there are mainly two forms of *gvp* genes, namely *p‐vac* and *c‐vac* (*p* stands for plasmid and *c* stands for mini‐chromosome). *H. salinarum* str. PHH1 and R1 contain both *p‐vac* and *c‐vac*, while *H. salinarum* strains of PHH4, SB3, GN101 and GRB only contain *c‐vac*. For example, cyanobacteria generally shape like a cylinder terminated by conical caps and have a diameter of about 45–120 nm and length from 100 nm to over 1 µm, which is dependent on *c‐vac*. Most of the GVs in *H. salinarum* shape like spindle resulting from *p‐vac*.^[^ ^77^ ^]^ The synthesis of GVs is also environmentally dependent. It is affected by oxygen concentration, light intensity, salt supply, and temperature. The growth of *H. salinarum* GVs is induced by low temperature (15 °C). High salinity (>20% weight per volume) leads to the generation of *Haloferax mediterranei* GVs. Light intensity meditates the GVs of photosynthetic bacteria.^[^ ^73^ ^]^

**Table 1:** Characteristics and functions of Gvps. Adapted with permission.^[^ ^75^ ^]^ Copyright 1997, Oxford Academic

Gvps	Molecular weight [kDa]	Hydrophobicity	Isoelectric point	Function
GvpA	≈7–8	Hydrophobic^[^ ^78^ ^]^	4.0	Major structural protein forming ribs
GvpB	≈8	Hydrophobic^a)^	4.0	Structural protein associated with minor cylindrical form
GvpC	≈31–42	Hydrophilic^[^ ^78^ ^]^	3.6	Outer structural protein stabilizing structure, assisting in growth, and determining shape
GvpD	≈59	Hydrophilic^a)^	4.2	Negative regulatory protein^[^ ^77^ ^]^
GvpE	≈21	Hydrophilic^a)^	4.1	Regulatory protein and transcriptional activator^[^ ^74^ ^]^
GvpF	≈24	Hydrophilic^a)^	4.0	Possible minor structural protein or assembly protein
GvpG	≈10	Hydrophilic^[^ ^74^ ^]^	4.1	Possible minor structural protein or assembly protein
GvpH	≈20	Hydrophilic^[^ ^74^ ^]^	3.9	Possible minor structural protein or assembly protein
GvpI	≈16	Hydrophilic^a)^	10.8	Related with GV length^[^ ^74^ ^]^
GvpJ	≈12	Hydrophobic^[^ ^73^ ^]^	3.7	Partner of GvpA at specific locations in GV wall^[^ ^73^ ^]^
GvpK	≈13	Hydrophilic/hydrophobic^a)^, ^b)^	3.9	Possible minor structural protein or assembly protein
GvpL	≈32	Hydrophilic^a)^	4.2	Possible minor structural protein or assembly protein
GvpM	≈9	Hydrophobic^[^ ^74^ ^]^	4.1	Partner of GvpA at specific locations in GV wall^[^ ^73^ ^]^
GvpN	≈39	Hydrophilic^a)^	4.9	Assembly protein containing a nucleotide‐binding motif^[^ ^73^ ^]^ and related with GV size^[^ ^74^ ^]^
GvpO	≈12–15	Hydrophilic^a)^	≈4.0–4.2	Possible regulatory protein

^a)^ Predicted through grand average of hydropathicity (GRAVY) from ExPASy;

^b)^ Hydrophilic in some organisms such as *H. salinarum* and *H. mediterranei*, and hydrophobic in other organisms such as *Nostoc sp*. and *Anabaena flos‐aquae*.

Apart from the innate genetic differences among hosts, the mechanical and acoustic properties can be engineered by the modification of GvpC protein; thus, realizing harmonic response, collapse pressure, surface charge, targeting specificity, and fluorescent signals (Figure 6D).

### 4.2 Applications of Genetically‐Encoded GVs

Due to the unique physical properties, GVs have been identified as US reporters that are detectable at subnanomolar concentrations; and further, genetically engineered as acoustic biosensors to track cellular activities.

**Figure 7:**
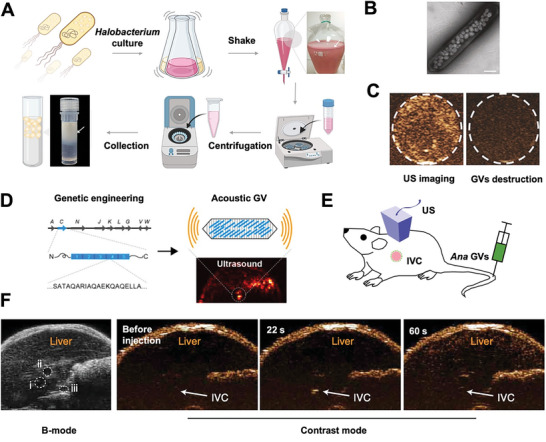
A) Scheme of the harvesting and purification procedure of GVs from *Halobacterium*. B) TEM of *Halobacterium* GVs. C) US contrast images of HEK293 cells that take up p‐DNA@GVs before (left) and after (right) acoustic destruction. Contrast signals disappear post intracellular GVs destruction. (A–C) Reproduced under the terms of the Creative Commons CC‐BY license.^[^ ^82^ ^]^ Copyright 2023, The Authors. Published by Springer Nature. D) Scheme of acoustic GVs by genetical modification of GvpC protein. E) Schematic illustration of US contrast imaging post injection of *Ana* GVs. (D,E) Adapted with permission.^[^ ^76^ ^]^ Copyright 2018, American Chemical Society. F) US contrast imaging of liver post injection of *Ana* GVs. IVC, inferior vena cava. Adapted with permission.^[^ ^17^ ^]^ Copyright 2017, Springer Nature.

A recent application reported by Yan and coworkers showed that *Halobacterium* GVs took up by HEK293 cells could remain stable post 24 h and collapsed under acoustic irradiation in a controllable manner. The process was monitored by real‐time US imaging (**Figure** **7**A–C). More importantly, the authors used the GVs to deliver E‐cadherin nuclear gene with US controllability, eventually inhibiting tumor invasion and metastasis inhibition. This study provided a useful strategy to genetically regulate cells behaviors and investigate the underlying mechanisms with assistance of GVs and US technology. Some similar experiments showed that engineered GVs responded to sound pressure with nonlinear mechanical deformation, which allowed them to be imaged selectively by tailored amplitude modulation strategy.^[^ ^79^ ^]^ Under a certain threshold of acoustic pressure, ligand‐modified GVs would site‐specifically burst to produce cavitation effect that could be used to localize cells.^[^ ^80^ ^]^ It has been tested that second and third harmonic signals in GVs could be detected. By transmitting 6 MHz US pulse, 12 MHz, and 18 MHz, harmonic signals can be observed in *Halo* GVs. Furthermore, GVs with different shapes and sizes have distinct US responses and critical collapse pressures, making it possible to distinguish difkinds of GVs through “serial collapse” imaging. For example, the critical collapses of *Halo* and *Ana* GVs are ≈70–150 kPa and ≈440–605 kPa, respectively.^[^ ^81^ ^]^

Another particularly promising advantage of GVs is that their physical and biochemical properties can be genetically modified. Lakshmanan et al. prepared biogenic GVs to achieve high‐performance US imaging. GVs were isolated from *A. flos‐aquae* (*Ana*) and *H. salinarum* (*Halo*) cells and further purified with buoyancy‐assisted techniques. Through chemical functionalization and genetic engineering, these GVs achieved enhanced US imaging and multiplexed imaging, which proved the possibility that Gvp genes could be developed as reporter genes for non‐invasive imaging at the molecular and cellular levels^[^ ^17^ ^]^ (Figure 7D–F).

Acoustic reporter genes (ARGs) allow enhanced US contrasts. For example, Hurt et al. genetically encoded ARGs that enabled real‐time US imaging of in situ tumor colonization at a depth greater than 1 cm and gene expression in tumor‐homing therapeutic bacteria. In addition, ARGs allowed long‐term monitoring of gene expression and tumor growth on breast cancer mouse models.^[^ ^83^ ^]^ Further investigations realized the expression of ARGs in mammalian cells and living animals by co‐transfecting total nine GV genes in the form of plasmid encoding GvpB, plasmid containing eight P2A‐tolerant GV genes, and “booster” plasmid containing bottleneck genes. Results showed that this mammalian ARG allowed high resolution imaging at cellular level which US previously could not achieve.^[^ ^84^ ^]^ GVs were also used to improve contrast in functional US imaging of the mouse brain to obtain amplified neuroimaging signals.^[^ ^85^ ^]^

Another application of GVs is serving as US biosensors of cellular enzyme activities. Lakshmanan and coworkers showed that protease‐activatable GVs could be generated through engineering variants of GvpC incorporating amino acid sequences which were recognized and acted on by specific proteases. The constitutively active tobacco etch virus endopeptidase, the calcium‐dependent mammalian protease calpain, and the processive bacterial protease ClpXP responsive GVs were engineered to reveal proteases’ activity under US radiation and to demonstrate the ability of biosensor imaging in vitro, in living bacteria and in mouse gastrointestinal tract.^[^ ^86^ ^]^

In summary, due to their modifiable properties by surface GvpC protein modification and chemical functionalization, GVs have proved to be useful as US contrast agents and acoustic biosensors. Further investigations will focus on engineering of GVs shell and optimizing their capability for in vivo imaging and biosensing.

## 5 MRI Contrast Agents

MRI can be used for multi‐sequence and multi‐parameter imaging to form contrast images of different signals including T_1_, T_2_, proton density, chemical shift, magnetization transfer, perfusion, and diffusion. However, conventional metal‐based contrast agents could cause adverse effects, such as spurious hypocalcemia, high gadolinium deposition in bone, and nephrotoxicity.^[^ ^87^ ^]^ In addition, exogenous MRI contrast agents have intrinsic limitations to function as biomolecular tools. Biogenic MRI contrast agents would help to overcome these problems. Apart from biogenic T_1_ and T_2_ agents, such as metalloprotein and ferritin, many other biogenic agents, including proton exchangeable agents for CEST,^[^ ^88^ ^]^ GVs for hyperCEST,^[^ ^89^ ^]^ and AQP for DWI,^[^ ^90^, ^91^ ^]^ have been discovered and developed for cellular and molecular research.

### 5.1 T_1_ Agents: Metalloprotein‐Based Agents

Naturally‐occurring metalloproteins tend to contain Cu^2+^, Mn^2+^, Mn^3+^, Fe^2+^, and Fe^3+^ ions, which make them paramagnetic; and thereby, detectable by MRI techniques in similar mechanisms of gadolinium‐based MRI. One particular advantage of metalloproteins is that they can be artificially engineered to improve their relaxivity by means of site mutation and metal substitution.^[^ ^92^, ^93^ ^]^ MRI of metalloproteins also promotes the understanding of metal ion metabolism in living systems.^[^ ^94^ ^]^

#### 5.1.1 Hemoglobin

**Figure 8:**
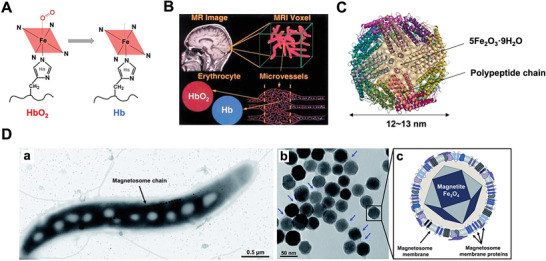
A) Chemical structures of HbO_2_ and Hb. B) The Hb oxygenation status is used for MRI. Adapted with permission.^[^ ^121^ ^]^ Copyright 1998, Springer Nature. C) The structure of ferritin consisting of 5Fe_2_O_3_·9H_2_O and polypeptide chain. D) Morphology of magnetosome. TEM of a representative cell of *M. gryphiswaldense* (left). TEM of isolated magnetosomes. Magnetosomes are made of a core of pure magnetite (Fe_3_O_4_) which is enveloped by magnetosome membrane (middle). Schematic diagram of a single magnetosome nanoparticle. The magnetosome membrane harbors a series of magnetosome specific proteins (right). Reproduced under the terms of the Creative Commons CC‐BY license.^[^ ^122^ ^]^ Copyright 2021, The Authors. Published by Royal Society of Chemistry.

Hemoglobin (Hb) contains four polypeptides forming globin and each polypeptide binds to a heme‐Fe group (ferroprotoporphyrin), making Hb an oxygen‐sensitive contrast agent (**Figure** **8**A,B).^[^ ^95^ ^]^ Hb can shift between diamagnetic oxyhemoglobin (HbO_2_) and paramagnetic deoxyhemoglobin under different oxygen concentration, producing blood oxygenation level‐dependent image contrast.^[^ ^96^ ^]^ It is a powerful tool to study neuroscience,^[^ ^97^ ^]^ tumor hypoxia,^[^ ^98^ ^]^ perfusion and ischemia,^[^ ^99^ ^]^ and skeletal muscle,^[^ ^100^ ^]^ among others.

#### 5.1.2 Cytochrome P450

Similar to Hb, cytochrome P450 (CYP450) contains a heme‐Fe group producing T_1_ contrast with *r*_1_ around 1.4 mm ^−1^ s^−1^ at 4.7 T. Shapiro et al. reported the engineering of neurotransmitter dopamine's sensor generated from the heme domain of bacterial CYP450‐BM3 (BM3h), which showed a high affinity and responsive signal drop toward dopamine.^[^ ^101^ ^]^ This sensor had further been used to investigate neurotransmitter release and uptake in the brain.^[^ ^102^, ^103^ ^]^ The Fe atom of CYP450 heme‐Fe group could be substituted artificially with higher‐spin metals, such as manganese, to augment the relaxivity. Lelyveld and colleagues developed manganese (III)‐containing BM3h protein with higher electron spin than their native ferric iron counterparts,^[^ ^93^ ^]^ which showed improved T_1_ relaxivities and offered a new strategy to engineer effective protein‐based sensors for molecular MRI.

#### 5.1.3 Divalent Metal Transporter 1

Divalent metal transporter 1 (DMT1), discovered by Gunshin et al. in 1997, plays a key role in transporting divalent cations including Mn^2+^, Cu^2+^, Fe^2+^, and Zn^2+^.^[^ ^104^ ^]^ It can be genetically engineered, and thereby, site‐specially expressed in vivo to increase cell uptake of metal ions and augment cellular signal of manganese‐enhanced MRI for observing brain tumor cells^[^ ^105^ ^]^ and stem cells.^[^ ^106^ ^]^

### 5.2 T_2_ Agents: Ferritin and Magnetosome

#### 5.2.1 Ferritin

Ferritin and magnetosome are two common types of biogenic T_2_ agents. The comparison of the structures and MRI properties between ferritin and magnetosome is shown in **Table** **2**. Ferritin is one of the first biosynthetic superparamagnetic species used in T_2_WI, which is an iron storage protein in human body. Ferritin accumulates up to 4500 Fe (III) atoms in the form of 5Fe_2_O_3_·9H_2_O inside a protein shell and has a total molecular weight of about 480 kDa.^[^ ^107^ ^]^ Ferritin protein shell is formed by 24 polypeptide chains, whose inner diameter is ≈7–8 nm and outer diameter is ≈12–13 nm (Figure 8C). In vertebrates, each polypeptide chain contains two types of subunits, namely H chain (FTH) and L chain (FTL). H chain is associated with oxidase activity site that catalyzes the oxidation of two Fe (II) atoms; L chain is responsible for the formation of “cavity”^[^ ^107^, ^108^ ^]^ and is usually used as an MRI reporter gene.

**Table 2:** Comparison of the structures and MRI properties of magnetosome and ferritin. Adapted with permission.^[^ ^137^ ^]^ Copyright 2012, John Wiley and Sons

Biogenic T_2_ agent	Ferritin	Magnetosome
Species	Multiple organisms	MTB
Size [nm]	≈12–13	≈40–100
Crystal form	Hexagonal	Cubo‐octahedral
Iron form	5Fe_2_O_3_·9H_2_O	Fe_3_O_4_ /Fe_3_S_4_ (less commonly)
Vehicle	Protein shell	Lipid biolayer
*r*_2_/*r*_1_	8	19

Ferritin distributed in spleen and liver provides an *r*_2_ of ≈1.2–1.5 (mm Fe)^−1^ s^−1^ at 300 MHz.^[^ ^109^ ^]^ It was indicated that differential iron levels might be adequate to offer MRI contrast.^[^ ^110^ ^]^ More intriguingly, the development of gene editing technology has paved a way for ferritin to be highly expressed in and to specifically target to cells as an MRI reporter that simultaneously monitors the anatomical location and viability of cells. Ferritin has been proved useful for investigation on stem cell transplantation and tracking,^[^ ^111^, ^112^, ^113^ ^]^ neural cell migration,^[^ ^114^ ^]^ cell differentiation,^[^ ^115^ ^]^ immune cell tracking,^[^ ^116^, ^117^ ^]^ brain visualization,^[^ ^118^ ^]^ tumor growth,^[^ ^119^ ^]^ and microstructural changes.^[^ ^120^ ^]^

#### 5.2.2 Ferritin‐Related Genes and Proteins

Although the final cellular contrast signal is provided by ferritin‐encapsulated iron, several molecules in iron metabolism pathway have been used to enhance cellular iron accumulation, including transferrin receptor, DMT1, T‐cell immunoglobulin, and mucin domain containing protein 2 (TIM2).^[^ ^110^ ^]^ Transferrin receptor is the main cellular protein responsible for iron uptake.^[^ ^110^ ^]^ A study revealed that in a transgenic organism, FTH generated MRI contrast signal through compensatory upregulation of transferrin receptor, which resulted in enhanced storage of cellular iron in ferritin‐bound form.^[^ ^123^ ^]^ As expected, it realized higher MRI performance when ferritin and transferrin receptor were transduced in combination than the expression of FTH or transferrin receptor alone.^[^ ^124^ ^]^ Some types of cells including oligodendrocytes,^[^ ^125^ ^]^ B lymphocytes, and T lymphocytes^[^ ^126^ ^]^ employ TIM2 as delivery molecule, which is a receptor for FTH and its endocytosis.^[^ ^126^ ^]^ Recently, a study showed that mice implanted with TIM2‐expressing cells in xenograft tissue shortened T_2_ after ferritin injection, demonstrating that TIM2 had the potential to serve as an MRI reporter protein.^[^ ^127^ ^]^

#### 5.2.3 Magnetosome

Magnetosome is another important biogenic T_2_ agent, a membranous structure present in magnetotactic bacteria, which was discovered by Blakemore in 1975.^[^ ^128^ ^]^ Magnetosome has a diameter of ≈40–100 nm and a core formed of either magnetite (Fe_3_O_4_) or, less commonly, pyrite (Fe_3_S_4_) through biomineralization (Figure 8D).^[^ ^129^ ^]^ It shows stable monocrystalline structure, uniform particle size (if produced by same species of magnetotactic bacteria), and permanent magnetism. In addition, magnetosome exhibits the features of phospholipid bilayer biofilm structure, allowing specific chemical modification. More importantly, it has been proved to exhibit a higher T_2_ relaxivity and lower toxicity than commercial agents ferumoxide.^[^ ^130^, ^131^, ^132^ ^]^

The biomineralization of magnetosomes involves about 30 genes clustered in a genome region called magnetosome island, which contains magnetosome membrane gene and magnetic‐particle membrane specific gene that could perform as MRI reporter genes.^[^ ^133^ ^]^ With the development of antibody, peptide, and surface modification materials, magnetosome has extensive applications in MRI for tumor. For example, magnetosome nanoparticles functionalized with EGFR/HER2 targeting peptide could be used to detect breast cancer.^[^ ^134^ ^]^ Magnetosome modified with anti‐EGFR produced higher signal‐to‐noise ratio than HSA‐coated (HAS: human serum albumin) iron oxide nanoparticles modified with anti‐EGFR.^[^ ^135^ ^]^ Likewise, magnetosomes decorated with RGD peptide could specifically target glioblastoma U87 cells.^[^ ^136^ ^]^

### 5.3 Proton Exchangeable Agents for CEST MRI

**Figure 9:**
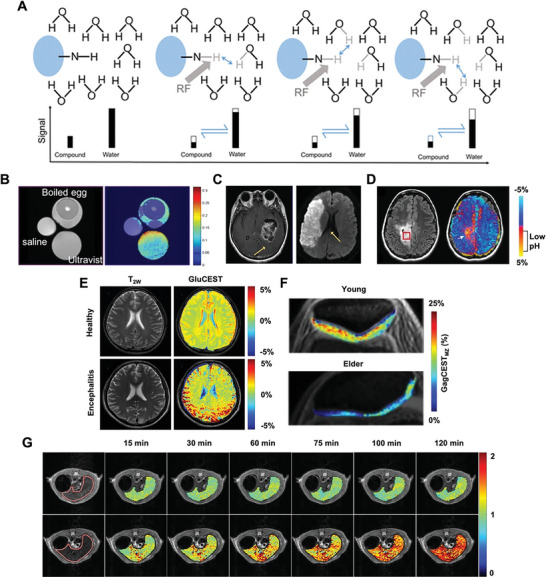
A) Principle of CEST: the target substance at limited concentration is saturated by a radiofrequency. The reduced signal of the substance is shown as the shortened black bar. Then, the saturated hydrogen proton is transferred to water molecule to return an unsaturation, which occurs many times to cause amplified water signal reduction. B) Comparison between conventional MRI (left) and CEST MRI (right). C) ATP MRI for brain tumor (left) and stroke (right). (A–C) Reproduced under the terms of the Creative Commons CC‐BY license.^[^ ^139^ ^]^ Copyright 2016, The Authors. Published by Springer Nature. D) Fluid attenuated inversion recovery imaging of brain tumor shows CEST asymmetry at 3.0 ppm (left). The corresponding pH‐weighted MRI shows lesion localization in low pH regions (right). Adapted with permission.^[^ ^138^ ^]^ Copyright 2017, Wiley. E) GluCEST MRI of human brain using the 3.0 T MR system. In a healthy volunteer, both T_2_W imaging and GluCEST MRI could distinguish white matter and gray matter. In contrast, in an encephalitis patient, GluCEST imaging could additionally clearly show the left parietal cortex and subcortex lesion. Reproduced under the terms of the Creative Commons CC‐BY license.^[^ ^140^ ^]^ Copyright 2020, The Authors. Published by Frontiers. F) GagCEST MRI of patellar cartilage in a young volunteer and in an elderly patient with knee pain, respectively. Adapted with permission.^[^ ^141^ ^]^ Copyright 2016, Wiley. G) Dynamic monitoring of glycogen metabolism in mice liver infused with saline (top) and glucagon (bottom). Reproduced under the terms of the Creative Commons CC‐BY license.^[^ ^142^ ^]^ Copyright 2022, The Authors. Published by ScienceDirect.

Standard MRI contrasts work depending on the spin relaxation rates of different body tissues under a static magnetic field and various radiofrequency pulses. CEST is a novel MRI technique that allows detection of certain compounds with limited concentrations, which is unable to enhance the contrast of conventional MRI, achieving unprecedented sensitivity at the molecular level.^[^ ^138^ ^]^ The principle of CEST is shown in the schematic diagram below (**Figure** **9**A). Generally, tissues show no net magnetization, namely signal reduction on MR images, if in a state of saturation. For example, fat suppression technique uses saturation at the fat frequency so that the fat signal is removed from the subsequent imaging. Similarly, magnetization in CEST is transferred from other molecules (usually at very low concentration) to water molecules (abundant in human body); so that, the saturation effect on the targeted species can be observed on water instead.^[^ ^138^ ^]^ For example, T_2_‐weighted images show limited differentiation among saline water, egg white, and iopromide solution Ultravist (contain amide groups that resonate at 4.2 and 5.6 ppm w.r.t. water), whereas significant contrast of iopromide and egg white at 4.2 pm is observed on CEST MRI.

The prerequisite for this transfer of saturation effect is that the target compounds must be able to exchange protons with the surrounding water molecules. There are abundant endogenous biomolecules capable of exchanging protons with water molecules, including small molecular substances (urea, ammonia water, and glucose), large molecular polymers (poly‐lysine, poly‐arginine, and mucopolysaccharide), and main metabolites (such as protein, polypeptide, and creatine). With appropriate CEST rate, these CEST contrast agents can generate remarkable signals.^[^ ^143^ ^]^ In addition, the chemical shifts of CEST peaks are related to their hydrogen atoms’ chemical environments. Generally, amide proton and amine proton of mobile proteins and peptides show CEST peak at 3.5 and 3.0 ppm, respectively; hydroxyl proton of glycosaminoglycan (GAG) displays CEST peak ranging from 0.9 to 1.9 ppm, and guanidinium proton of creatine exhibits CEST peak at 1.8 ppm.

As to the proton exchange rate, adequate exchange interval (faster than T_1_ and T_2_ relaxation) is required to achieve selective proton irradiation for efficient saturation transfer.^[^ ^144^, ^145^ ^]^ Many alcohols and amines in alkali condition are not amenable to CEST because proton exchange rate on their functional groups is often too fast.^[^ ^146^ ^]^ Amide proton typically has a chemical exchange rate of ≈10–200 Hz,^[^ ^147^ ^]^ while hydroxyl proton has a faster chemical exchange rate of over 1000 Hz. In addition, chemical exchange rate is not only related to chemical groups but also affected by external environment, such as pH. The well‐known amine/amide concentration‐independent detection (AACID) is based on environmental pH.^[^ ^148^ ^]^

Based on the contrast agents, CEST MRI can be classified into amide proton transfer (APT) MRI, amine CEST MRI, GluCEST MRI, GagCEST MRI, GlycoCEST MRI, CrCEST MRI, and so on. These CEST MRI tools can be used to evaluate body's physiological and pathological conditions, such as muscle physiology, lymphedema, osteoarthritis, brain ischemia, nervous system diseases, and solid tumors. As shown in **Table** **3**, CEST MRI contrast agents and their corresponding characters, mechanisms, and applications are summarized.

**Table 3:** Endogenous CEST MRI contrast agents and their properties and applications

Exchangeable proton	CEST peak [ppm]	Exchange rate [Hz]	Contrast agent	Application	Mechanism
Amide proton (—CONH—)	≈3.5–3.8 ^[^ ^147^, ^192^ ^]^	≈10–300^[^ ^147^ ^]^	Proteins	Cerebral ischemia^[^ ^162^, ^163^ ^]^	Decreased pH/exchange rate
			Interstitial proteins	Lymphedema^[^ ^193^ ^]^	Interstitial protein accumulation
			Overexpressed proteins	Brain tumor^[^ ^194^, ^195^, ^196^, ^197^, ^198^, ^199^, ^200^ ^]^	Decreased exchange rate caused by acidic TME^a)^
			Proteins	Multiple sclerosis^[^ ^201^, ^202^ ^]^	Accumulation of mobile proteins in normal‐appearing white matter
			Deposited proteins	AD^[^ ^203^ ^]^	Abnormal protein deposition
Amine proton (—NH_2_)	3.0^[^ ^164^ ^]^	5500	Overexpressed proteins	Gliomas^[^ ^165^ ^]^	Increased protein concentration
			Proteins	Cerebral ischemia^[^ ^148^ ^]^	AACID^[^ ^148^ ^]^
			Glutamate	Neurological disorders^[^ ^166^, ^167^, ^168^, ^169^ ^]^	Glutamate concentration
Hydroxyl proton (—OH)	≈0.9–1.9^[^ ^175^ ^]^	>1000	Gag	Osteoarthritis^[^ ^175^, ^204^, ^205^, ^206^, ^207^, ^208^ ^]^	The loss of gag as an early indicator of osteoarthritis
	≈0.8–2.2 ^[^ ^181^, ^182^ ^]^	>1000	Glucose	Tumor detection^[^ ^181^, ^182^ ^]^	Over‐uptake of glucose in tumor cells
	≈0.5–1.5^[^ ^183^ ^]^	>1000^[^ ^183^ ^]^	Glycogen	Monitoring glycogen^[^ ^183^ ^]^	Hepatic glycogen transport
Guanidinium proton	≈1.8–2^[^ ^191^ ^]^	≈850–1050^[^ ^209^ ^]^	Creatine	Creatine detection^[^ ^210^, ^211^, ^212^, ^213^ ^]^	Creatine concentration

^a)^ TME: tumor microenvironment.

#### 5.3.1 APT MRI

APT MRI signal relies on the concentration and exchange rate of amide protons and further depends on the protein concentration and pH within tissues. Therefore, APT MRI can be used to detect pathological changes associated with overexpressed proteins or decreased pH, such as cerebral ischemia, brain tumor, and neurodegenerative diseases. In malignant tumors, overexpressed proteins play an important role in APT signal enhancement.^[^ ^149^ ^]^ In clinical, APT MRI is mainly applied to grade meningiomas^[^ ^150^ ^]^ and gliomas^[^ ^151^, ^152^, ^153^ ^]^ and to identify high grade glioma from solid metastasis and primary central nervous system (CNS) lymphoma.^[^ ^154^, ^155^ ^]^ APT MRI is also able to diagnose neurodegenerative diseases, including Parkinson's disease (PD)^[^ ^156^ ^]^ and Alzheimer's disease (AD),^[^ ^157^ ^]^ which are associated with abnormal protein deposition in CNS.

For cerebral ischemia at the initial stage, the signal level depends on the change of pH value due to anaerobic respiration. For every drop of 0.5 pH units, the exchange rate of amide proton decreases by 50% and lesion area displays a low APT signal. Hence, APT MRI is able to differentiate ischemic and hemorrhagic cerebral infarction,^[^ ^158^ ^]^ subacute and acute intracranial hemorrhage,^[^ ^159^, ^160^ ^]^ and ischemic penumbra and benign oligemia.^[^ ^161^, ^162^, ^163^ ^]^ For comparison, an increased level of APT can be observed in brain tumor, whereas decreased APT can be seen in stroke (Figure 9C).

#### 5.3.2 Amine CEST MRI

Amine CEST MRI is particularly useful as an amine‐ and pH‐sensitive imaging technique, which could target high concentration of amino acids with exchangeable amine protons and reduced extracellular pH.^[^ ^164^, ^165^ ^]^ It almost shows no signals under alkaline conditions, while in acid environments, resonance frequency at 3.0 ppm can be detected by MRI. In clinical trials, amine CEST MRI was used to detect gliomas cancer.^[^ ^164^, ^165^ ^]^ In AACID, resonance frequency at 3.5 ppm of amide protons is observed to measure absolute pH on mouse model by ratio metric technique.^[^ ^148^ ^]^

#### 5.3.3 GluCEST MRI

Glutamate (Glu) is a major neurotransmitter in the brain, the depletion of which will cause an imbalance in the dopaminergic system, resulting in progression of psychoses. On the contrary, over‐secretion of Glu leads to excitotoxic effects on the N‐methyl‐D‐aspartic acid receptor, which is related to AD and Huntington's disease. Therefore, the mapping of Glu could help gain deeper insights into the understanding of neuropsychiatric disorders. The corresponding studies including mapping Glu in the human brain^[^ ^166^, ^167^ ^]^ and spinal cord^[^ ^168^ ^]^ have been conducted with Glu CEST MRI. Glu imaging of lobe epilepsy,^[^ ^169^ ^]^ psychosis,^[^ ^170^ ^]^ and multiple sclerosis^[^ ^171^ ^]^ was also implemented on patients. Other CNS diseases, such as AD,^[^ ^172^ ^]^ PD,^[^ ^173^ ^]^ and Huntingdon's disease,^[^ ^174^ ^]^ have been investigated on animal models with Glu CEST MRI. Of note, clinical Glu CEST MRI studies have only been applied in ultrahigh magnetic field strength of 7 T because quantification of such specific neurotransmitter within a required accuracy remains limited at standard 3 T field strength.^[^ ^166^, ^167^, ^168^ ^]^

#### 5.3.4 GagCEST MRI

GagCEST was first reported to assess glycosaminoglycan (Gag) concentration in vivo in 2008.^[^ ^175^ ^]^ Osteoarthritis is a common chronic disease beginning with cartilage degeneration. In its early stage, the composition of extracellular matrix changes, such as the loss of proteoglycan (PG) molecules, which is mainly based on Gag.^[^ ^176^ ^]^ GagCEST technology is the latest cartilage component quantitative technology that can realize the non‐invasive and accurate quantification of PG/GAG components in cartilage extracellular matrix. GagCEST MRI detects the changes in the CEST effect at hydrogen proton frequency of Gag, facilitating super early diagnosis with high sensitivity and signal consistency.^[^ ^177^, ^178^ ^]^

#### 5.3.5 GlucoCEST MRI

Glucose is an important source of energy for humans as well as a necessity for tumor growth and proliferation. Therefore, GlucoCEST MRI can be used to detect tumors by measuring dynamic distribution of glucose, which has been applied to study glioma^[^ ^179^ ^]^ and head and neck cancer.^[^ ^180^ ^]^ Practically, D‐glucose (about 0.5–1.7 mmol/kg) is intravenously injected into patients; and then, saturated with a power of ≈1.6–2 µT. Last, it produces a wide CEST frequency spectrum between 0.8 and 2.2 ppm from the hydroxyl group at 3T or higher magnetic field strength.^[^ ^181^, ^182^ ^]^ More precise D‐glucose distribution mapping can be acquired by further comparing CEST spectrum obtained before and after the injection of D‐glucose.

#### 5.3.6 GlycoCEST MRI

Glycogen is a major energy source for regulating blood glucose level. Dysmetabolism of glycogen may cause many diseases, such as diabetes and insulin resistance. GlycoCEST MRI was reported to measure glycogen of perfused liver on mouse models by collecting signal between 0.5 and 1.5 ppm frequency range.^[^ ^183^, ^184^ ^]^ Recently, an experiment was conducted in the calf muscle of volunteers with real‐time simultaneous shim and motion correction.^[^ ^185^ ^]^

#### 5.3.7 CrCEST MRI

Creatine, generated from ATP synthesis, is a key cellular bioenergetic metabolite that reflects metabolic activity of tissues and organs.^[^ ^186^ ^]^ Many diseases show abnormal energy supply, leading to changes in creatine level, which are commonly seen in tumors,^[^ ^187^, ^188^ ^]^ heart dysfunction,^[^ ^189^ ^]^ and mitochondrial disorders.^[^ ^190^ ^]^ Hence, CrCEST MRI is able to detect those diseases based on creatine concentration changes. The specific saturating pulse frequency is guanidinium proton frequency at ≈1.8–2 ppm of creatine.^[^ ^191^ ^]^

### 5.4 AQP for DWI

In organisms, water is divided between cells and extracellular components. It diffuses relatively free in extracellular environments while experiencing “restricted diffusion” due to the restriction by surrounding media. As different diffusion properties exist in the human body, the direction of diffusion can indirectly reflect the characteristics and changes of various physiological and pathological progresses. For example, the diffusion becomes relatively restricted in high grade malignancies and acutely infarcted tissues due to the increase of intracellular proportion. DWI is thereby used to provide qualitative and quantitative information about the diffusion properties manner by measuring the signal intensity changes before and after applying diffusion sensitive gradient field.^[^ ^214^ ^]^ It is widely employed to evaluate molecular function and micro‐architecture of the human body. In addition to brain imaging,^[^ ^215^, ^216^ ^]^ DWI can also image breast cancer,^[^ ^217^ ^]^ prostate cancer,^[^ ^218^ ^]^ and hepatocellular carcinoma.^[^ ^219^ ^]^

Contrast in DWI can be enhanced by overexpressing water‐permeable channels to facilitate water exchange across the membrane. The most known protein for transmembrane diffusion is AQP,^[^ ^220^ ^]^ which was first discovered by Agre from erythrocyte membrane and was acknowledged by 2013 Nobel prize in chemistry. AQP is a kind of highly conserved tetrameric integral membrane protein located on the cell inner membrane and forming a “pore” on the surface of cell membrane that mediates the flow of water. Each identical subunit has a molecular weight of 28 kDa and consists of six *α* helices. The upregulation of AQP is often associated with diseases, which makes it a contrast agent for DWI.^[^ ^221^, ^222^, ^223^, ^224^, ^225^ ^]^ To date, there are many kinds of AQP subtypes, such as AQP1, AQP2, AQP3, AQP4, which have different expression levels and tissue distribution in human body. Among them, AQP4 is mostly reported to be related with various brain and neurological diseases,^[^ ^226^, ^227^, ^228^, ^229^ ^]^ such as cerebral and spinal edema,^[^ ^230^, ^231^, ^232^ ^]^ memory disease,^[^ ^225^ ^]^ cerebrovascular diseases,^[^ ^233^ ^]^ AD,^[^ ^234^, ^235^ ^]^ and malignant gliomas.^[^ ^236^ ^]^

### 5.5 Biogenic HyperCEST MRI Contrast Agents

#### 5.5.1 HyperCEST MRI

Hyperpolarization is a novel technique in MRI, which prepares highly magnetized nuclear and the MR‐visible signal by several thousand times, leading to significantly increased signal‐to‐noise ratio. Dissolution dynamic nuclear polarization (dDNP) is currently the most used method because of its flexibility in terms of marker molecules and high sensitivity. In dDNP, ^13^C‐labeled molecules typically^[1‐13C]^ pyruvate and ^13^C‐glucose are used to monitor their transport and downstream metabolites through ^13^C MRI chemical shift spectroscopy.

Based on the same mechanism, hyperpolarized noble gas can be inhaled into body, generating gas‐phase contrast, and enabling visualization of pulmonary structure and function. Among all the noble gases, ^129^Xe is the optimal option because its spin number is 1/2 and it is also stable and abundant in nature. A small part of ^129^Xe is further absorbed into the blood through pulmonary capillary blood and causes solution‐phase contrast. In 2006, Schröder et al. reported a pioneering study that binding ^129^Xe with specific organic compound could alter ^129^Xe chemical shift to generate MRI contrast at nanomolar concentration through the hyperpolarized CEST, termed HyperCEST.^[^ ^89^ ^]^ In practice, chemical host is first saturated with laser light; and then, transfers its energy to ^129^Xe to hyperpolarize ^129^Xe.

#### 5.5.2 GVs

Generally, ^129^Xe interacts with most proteins weakly. However, Shapiro and colleagues found that GVs could interact with hyperpolarized ^129^Xe to generate HyperCEST contrast signal at picomolar concentrations with peak saturation around 175 ppm up field from dissolved ^129^Xe,^[^ ^18^ ^]^ which paved a new path for genetically encoded reporters for HyperCEST.

In previous work, Shapiro et al. proved that the dissolved ^129^Xe signal at 195 ppm could be completely saturated with GVs concentration of 400 pm after exposing to 33.6 µT magnetic field for 6.5 s. GVs with diverse shape and size from different bacterial species possessed a unique chemical shift, which enabled multiplexed imaging. Furthermore, GV genes were transferred in *E. coli* to quantitatively observe gene expression as well as chemically functionalized to label breast cancer cells in vitro.^[^ ^18^ ^]^ So far, GVs have not been applied to animal studies as an MRI reporter gene for several reasons. First, it is a large multimeric protein edited by multiple genes so that it is hard to construct gene expression vector in eukaryotic systems; second, the transcription, translation, and protein folding between prokaryotes and eukaryotes are significantly different;^[^ ^237^, ^238^, ^239^ ^]^ third, Xe concentrations are supposed to be sufficient to detect 400 pm GVs using ^129^Xe HyperCEST for in vivo requirements.^[^ ^240^ ^]^ The translation of ^129^Xe HyperCEST MRI contrast agents from bench to bedside is still in progress. Further efforts need to be devoted to identifying modifiable and functional GVs with high imaging performance.

#### 5.5.3 *β*‐Lactamase

*β*‐lactamase, a single‐protein structure with a molecular weight of 29 kDa, can serve as a genetically encoded contrast agent for HyperCEST and provide a sensitive contrast in bacterial as well as mammalian cells. It induces an obvious saturation peak at 255 ppm through CEST interactions between ^129^Xe and allosteric site in *β*‐lactamase at a low concentration of 0.1 µm.^[^ ^241^ ^]^

Overall, compared to traditional gadolinium‐based MRI contrasts, BICAs for MRI are safer and more capable of targeting organs, sites of inflammation, and specific tumors. Despite the recent advances in new types of MRI BICAs, more research is required for future clinical translation, which, however, is often hindered by heterogeneity of endogenous biomarkers and the mechanisms of molecular compositions in diseases development and detection.

## 6 Bimodal Imaging Contrast Agents

With the rapid development of image processing and analysis technology, single‐modality medical imaging technology is undergoing a revolutionary transformation into integrated, multimodal, and cross‐scale imaging technology. Multimodal imaging technology can not only realize the extraction of comprehensive multi‐angle, multi‐parameter, and molecular‐level structure and function information of the same organism but also overcome the limitations of monomodal imaging, thereby improving the accuracy and efficiency of early detection of disease. Conventionally, bimodal contrast imaging is achieved by physical and chemical combination of different modal contrast agents, of which the procedure could be taxing and expensive. In contrast, gene‐encodable BICAs can be realized through genetic techniques which demonstrate a completely new strategy of bimodal imaging.

### 6.1 FL/PA Imaging Contrast Agents

As mentioned earlier, BphP‐based FPs can be used for both FL imaging and PAI^[^ ^242^ ^]^ but there is a trade‐off between these two imaging modes, either FL‐emission energy loss or non‐radiation energy loss occurring. BphP‐based FPs show different photophysical behavior in PAI than in FL imaging because PAI requires pulsed illumination and depends on signal generation via non‐radiative energy decay channels. This implies that rsFPs optimized for FL imaging may not be ideal for PAI.^[^ ^243^ ^]^

### 6.2 FL/MR Imaging Contrast Agents

The fusion and simultaneous expression of FP genes and ferritin genes can realize FL/MR bimodal imaging. Such dual reporter genes include ferritin‐DsRed,^[^ ^244^ ^]^ ferritin‐GFP,^[^ ^245^ ^]^ ferritin‐EGFP,^[^ ^246^, ^247^, ^248^, ^249^ ^]^ and so on. Besides, bacterial magnetosomes covalently modified with fluorescent dye could perform as bimodal contrast agents.^[^ ^250^ ^]^ Supercharged GFPs could also act as bimodal reporter genes for CEST MRI and FL imaging.^[^ ^251^ ^]^ Furthermore, Transferrin receptor‐luciferase gene was used as a bioluminescence/MRI dual reporter.^[^ ^119^ ^]^

### 6.3 US/MR Imaging Contrast Agents

GVs were first reported as a US contrast agent. It also can interact with hyperpolarized ^129^Xe to generate HyperCEST contrast, which makes it a natural contrast agent to achieve US/MRI bimodal imaging.^[^ ^252^ ^]^ Farhadi et al. described the recombinant expression of this nanostructure in *E. coli* and proved that GVs can generate US and HyperCEST MRI contrast at sub‐nanomolar concentrations.^[^ ^253^ ^]^ Other similar studies from the same laboratory also showed the bimodal imaging performance of GVs.^[^ ^254^, ^255^ ^]^

### 6.4 PA/MRI Imaging Contrast Agents

Melanin, mostly studied in PA imaging, can perform as the contrast agent for enhanced T_1_‐weighted MRI;^[^ ^256^, ^257^ ^]^ hence, it is possible for melanin to act as a PA/MR bimodal imaging contrast agent. In addition, many studies reported that its US signal could be enhanced several times through doping heavy metal elements, such as gold^[^ ^258^ ^]^ and gadolinium.^[^ ^259^ ^]^ For example, Lemaster et al. showed that Gd‐loaded melanin nanoparticles exhibited up to a 40‐fold enhanced photoacoustic signal intensity compared to melanin alone, displaying a better PA/MR bimodal imaging contrast performance.^[^ ^259^ ^]^ As a principle enzyme in melanin generation, tyrosinase has been used as a genetically‐encoded dual reporter molecule for both PAI and MRI.^[^ ^260^ ^]^

## 7 Summary and Outlook

In this review, the discoveries, properties, mechanisms, and applications of BICAs are discussed in four sections: FL imaging, US imaging, MRI, and bimodal imaging. Compared with exogenous contrasts, the advantageous feature of BICAs is genetical encodability, which lays the foundation for their real‐time expression, specific location, and tissue compatibility. For example, endogenous PpIX and melanin are excellent agents for contrast‐enhanced cancer imaging and subsequent guiding therapy. In addition, some MRI techniques utilize endogenous contrast agents including diamagnetic proteins for CEST, ferritin for T_2_WI, and AQP for DWI, which have already been applied in clinical practice.

In contrast, some contrast agents such as FPs for FL imaging and GVs for US imaging are not ready for clinical applications in human body.^[^ ^261^ ^]^ FPs, abundant in nature, have been well developed for biomedical research at cellular and tissue levels. Longer wavelength exposure is favorable to achieve in vivo imaging with deeper penetration. However, quantum yield of FPs in long wavelength region is relatively low. In addition, NIR FPs derived from BphP require BV, whereas BV enriches in liver and spleen, which makes it impossible for imaging of NIR FPs in BV‐insufficient tissues. Likewise, fluorogenic RNAs also need to bind with fluorophores. It requires high specificity and affinity of small fluorophore molecules to NIR FPs and fluorogenic RNAs to improve large scale imaging at the whole organism level.

The field of biomolecular US imaging looks for BICAs connecting ultrasonic physics with the dynamics and functions of biomolecules and cells to image cellular processes. The first and latest developed acoustic reporter gene is GV. It is still in the infancy, and future efforts, with the assistance of genetic engineering technology, are needed to improve the performance of GVs in biomedical research and US imaging. The gene clusters encoding GVs should be simplified to be efficiently transferred and expressed in a variety of cells. In addition, the relationship between Gvps and their acoustic properties should be further investigated to maximally distinguish their signals from background. Beyond US proteolytic sensors, it is anticipated to enable allosteric conformational changes rather than cleavage in GvpC to alter US contrast, thereby creating acoustic biosensors that respond reversibly to noncleaving enzymes, ions, and other signals of interest.^[^ ^86^ ^]^

As for MRI, its signal source is proton and the main detection methods are TWI, CEST, DWI, and HyperCEST. Compared to conventional chemically‐synthesized contrast agents, the development of BICAs, such as diamagnetic proteins and AQPs, is a remarkable progress extending the function of biomolecular MRI. MRI is born to address soft tissue contrast, allowing precise diagnosis in many diseases. However, in terms of neoplastic diseases, it requires not only distinguishing between benign and malignant tumor but also detailed staging and typing, which needs to combine MRI with metabolic information. Therefore, it is important to draw the spectra of multiple metabolites that correlate with neoplastic diseases. With the clinical application of BICAs, we presume that the evolution of future generations of metabolically‐related biogenic MRI contrast agents will play a greater role in diseases diagnosis, typing, and staging.

In conclusion, BICAs have been rapidly developed into useful tools for both laboratory research and clinical applications, while there are still challenges in the imaging depth, sensitivity, and signal contrast. We proposed possibilities and directions for future exploration to reach its full potential. With the advancement in cross‐disciplines such as genetic coding and engineering material in biomedical, BICAs hold promising potential to advance in vivo imaging research at cellar level and its clinical translations.

## Conflict of Interest

The authors declare no conflict of interest.
